# Managing Type 2 Diabetes Mellitus through Periodical Hospital Visits in the Aftermath of the Great East Japan Earthquake Disaster: A Retrospective Case Series

**DOI:** 10.1371/journal.pone.0125632

**Published:** 2015-05-06

**Authors:** Yoshitaka Nishikawa, Yuji Fukuda, Masaharu Tsubokura, Shigeaki Kato, Shuhei Nomura, Yasutoshi Saito

**Affiliations:** 1 Department of Internal Medicine, Soma Central Hospital, 3-5-18 Okinouchi, Soma, Fukushima, Japan; 2 Department of Radiation Protection, Soma Central Hospital, 3-5-18 Okinouchi, Soma, Fukushima, Japan; 3 Division of Social Communication System for Advanced Clinical Research, the Institute of Medical Science, University of Tokyo, 4-6-1 Shirokanedai, Minato-ku, Tokyo, Japan; 4 Department of Epidemiology and Biostatistics, School of Public Health, Imperial College London, Norfolk Place, London, United Kingdom; University of Tolima, COLOMBIA

## Abstract

**Aims:**

To assess the impact of the Great East Japan Earthquake Disaster on daily diabetes practice and to determine the feasibility of controlling type 2 diabetes mellitus in an outpatient department.

**Methods:**

We retrospectively reviewed the data on disaster-affected patients with type 2 diabetes who periodically attended outpatient department of Soma Central Hospital. There were 767 patients with type 2 diabetes mellitus in total. The primary outcome measure was the change in HbA1c.

**Results:**

HbA1c levels of 58 patients with periodical hospital visits did not deteriorate after the disasters. Moreover, there observed no significant difference in the mean of HbA1c levels among all age and sex throughout the year. While several changes in diabetes medication usage occurred, DPP4-inhibitor was the only oral diabetic agent that increased in frequency (+60%).

**Conclusions:**

Patients with type 2 diabetes who were managed with periodical hospital visits did not show significant deterioration in HbA1c levels.

## Introduction

Diabetes mellitus is one of the most common chronic diseases globally. With an estimated 366 million afflicted individuals in 2011, the worldwide prevalence is rising and expected to reach 522 million by 2030 [1]. Type 2 diabetes, the most prevalent type, has been demonstrated to be associated with lifestyle factors as well as genetic predisposition [2]. As diabetes progresses, the imbalance between insulin demand and supply becomes more pronounced, resulting in the broad fluctuation of blood glucose levels [3]. As a consequence of inadequate glycemic control, diabetic patients are at risk for developing serious complications such as coronary artery disease, stroke, diabetic feet, renal failure, blindness and neuropathy [4]. Therefore, maintaining glycemic control is essential for preventing fatal complications and improving the long-term prognosis of patients [5].

However, various factors and conditions, such as physical inactivity, excessive caloric intake, and mental stress, can make glycemic control difficult for patients with type 2 diabetes in daily medical practices. A major disaster represents one of the most challenging conditions imposing several risk factors for exacerbation in diabetes. Prior studies have shown that diabetes mellitus and other chronic diseases may be exacerbated in the aftermath of a major disaster [6–8] through various pathways, such as drug discontinuation, lifestyle changes, and stresses [9–12]. However, little information exists on how to maintain diabetes control in the early to mid-term period following a disaster, particularly during the first year.

**Fig 1:**
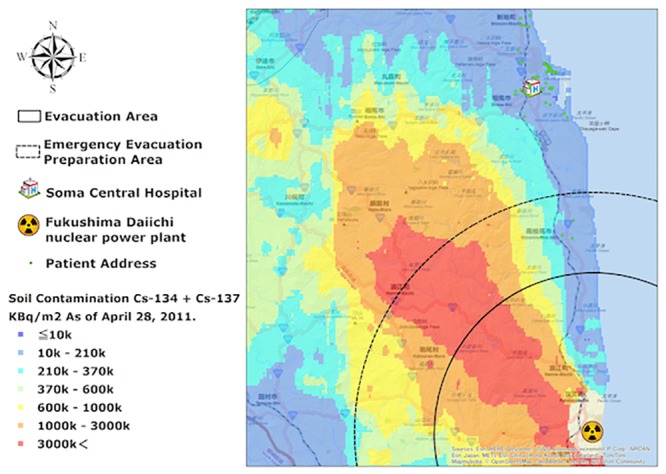
Map of north coastal area of Fukushima prefecture with level of soil contamination in April 2011. Patients’ locations, except for three patients from Miyagi, SCH and Fukushima Daiichi nuclear power plant are plotted on the map of Fukushima. Soma is a city located in the coastal area of Fukushima Prefecture affected by the Great East Japan Earthquake, tsunami, and the Fukushima Nuclear Disaster. Soma has a population of 36,000, of which about 26.9% are aged ≥65 years. The tsunami was recorded to have exceeded 9 m and spread 4 km inland into the city. Moreover, Soma is located about 40 km north of the Fukushima Daiichi nuclear power plant. The mountainous region of the city was contaminated with radiation due to the Fukushima Nuclear power plant incident. Four-hundred sixty-two people were killed, and more than 2,000 people were evacuated to temporary housing due to the series of disasters. Copyright © 2015 Esri, HERE, DeLorme, USGS, Intermap, iPC, NRCAN, Esri Japan, METI, Esri China (Hong Kong), Esri (Thailand), MapmyIndia, TomTom, © OpenStreetMap contributors, and the GIS User Community. All rights reserved.

On March 11, 2011, Japan experienced unprecedented catastrophic disasters in the form of the Great East Japan Earthquake followed by a tsunami and the Fukushima Daiichi nuclear power plant incident [13]. Located on the coast of the Fukushima Prefecture, Soma City is situated approximately 40 km north of the power plant and is the closest major city (Fig 1). Since the time preceding the disasters, the population residing in the city and surrounding towns has been largely vulnerable due to the chronic shortage of medical staff, advancing age, and geographical restrictions on rural living. Although the residents were exposed to minimal levels of radiation [14,15], the city suffered enormous damage from the tsunami, including 460 immediate deaths [16]. There are still 2,000 evacuees dwelling in temporary housing [16]. Additionally, several hospitals in the area were forced to close shortly after the disasters due to staffing shortages. As such, Soma Central Hospital (SCH) was the only hospital in the city that was able to offer and sustain a diabetes outpatient department following the disasters.

In the aftermath of these disasters, the affected areas have endured great difficulties in managing patients with type 2 diabetes. However, little is known about the optimal strategy for managing these patients during the early- to mid-term period following a major disaster. To assess the impact of the Great East Japan Earthquake Disaster (GEJED) on daily diabetes practice and the feasibility of controlling type 2 diabetes in an outpatient department, we retrospectively analyzed data from the SCH on patients with type 2 diabetes who were severely affected by the disaster. These findings provide useful information on controlling type 2 diabetes during and after a disaster through ongoing periodical visits.

## Material and Methods

### Study population

To evaluate the impact of GEJED on diabetes management practice, we retrospectively reviewed the data on disaster-affected patients with type 2 diabetes who periodically attended the SCH outpatient department. In this study, ‘disaster-affected’ patients referred to those who had obtained a disaster-victim certificate from the government. The certificates were issued to individuals whose houses had been fully or partially destroyed by the earthquake or tsunami or were located inside the emergency evacuation preparation area (the area between 20 and 30 km radius from the nuclear plant), and those whose households had lost the primary income earner due to the disasters. To assess the impact of the GEJED on daily diabetes practice, we followed only the patients with disaster victim certificates who had continued to visit the hospital periodically (PV (+)). We performed t-test between PV (+) and PV (-) groups, and between PV (+) and all the rest patients other than PV (+).

### Data collection and hospital visit schedule

To evaluate each patient’s diabetes profile, data on the following variables were extracted from the SCH database: hemoglobin A1c (HbA1c) levels (Japan Diabetes Society values), medication usage, demographic and clinical data, including gender, age, body weight, body mass index (BMI), blood glucose (BG), systolic blood pressure (SBP), diastolic blood pressure (DBP), total cholesterol (TC), low density lipoprotein cholesterol (LDL-C), high density lipoprotein cholesterol (HDL-C) and triglyceride (TG) levels. The Japan Diabetes Society measurements for HbA1c were converted to the international standard for HbA1c NGSP (National Glycohemoglobin Standardization Program) and IFCC (International Federation of Clinical Chemistry) [17].

Patients were followed monthly. HbA1c and BG levels, SBP, DBP, and BW were assessed at every visit. Other variables, including TC, LDL-C, HDL-C, and TG, were assessed at least once every three visits. Of note, during the period from March 11–16, 2011, blood testing was not feasible due to an interruption in power and water supply, and a shortage of test reagent.

Baseline characteristics were determined using the following methods. Data collected for the period from January to March 2011 provided the baseline pre-disaster measurements whereas data recorded during the period from July to September 2011 were designated as the post-disaster measurements. For patients who had multiple visits during a single period, we examined the average values for each parameter assessed. In order to evaluate the early to mid-stage impact of the disasters, the study period was limited to the interval between March 2009 and December 2011. Blood glucose levels were not included in the analyses because the assays were not necessarily performed on fasting specimens.

### Statistical analysis

Quantitative data were presented as the mean ± standard deviation (SD). Since metabolism and blood function may vary by age, the study population was divided into two groups: 1) a younger group, age <65 years, and 2) an elderly group, age ≥65 years. Potential underlying seasonal trends in HbA1c levels were expected; therefore, we compared the 2011 data with the average of the two preceding years (2009 and 2010) by season using a paired t-test. We stratified the total study population into four groups based on age group (<65 or ≥65 years) and sex. The seasonal effects were also assessed by visually inspecting a time-series of quarterly mean HbA1c levels and applying a B-spline-curve smoothing method. The numbers of medications prescribed for the entire study group before and after the disaster were compared. All p-values were two-sided with p-values ≤0.05 considered statistically significant. Statistical analyses of the baseline characteristics and changes in medication usage were performed using R version 3.0.2 (http://www.R-project.org/). The season-specific analyses of changes in HbA1c were performed using STATA/MP 13 (StataCorp LP; College Station, Texas, USA). The spatial data formatting were conducted using ArcGIS 10.2 (ESRI; Redlands, California, USA).

### Ethics approval

The ethics committee of the SCH granted approval for this study under authorization number 2014-B1. The ethics committee agreed that written consent from the participants was not required as this was a retrospective analysis of patient records. All the patients’ information was anonymized and de-identified prior to analysis.

## Results

### Basic characteristics of the study participants

The study population was comprised of 767patients who attended the SCH diabetes outpatient department three months before the disasters, of whom 170 had obtained a disaster-victim certificate from the Japanese government following the disasters. Excluding 112 patients without periodical visits, 58 eligible patients (PV (+)) representing 7.6% of all patients with diabetes mellitus at SCH were analyzed.

Data from 575 patients of all the rest of this study cohort are available from one year before the disaster (Table 1). The mean age was 63.3 years old (SD: 9.0) as compared with 67.3 (14.1) years old in the rest of all (p<0.01). HbA1c value was significantly higher in the study group than that in the rest [mean HbA1c, 7.3% (1.0) vs 7.0% (1.3), p<0.05]. Other health profiles were not significantly different. Data from 52 patients of all the disaster certificate patients without periodic visits (PV (-)) are available during one year before the disaster. No significant difference between PV (+) and PV (-) group is observed in age, sex, BG, T-Cho, TG, HDL, cLDL and HbA1c. The main causes of damage are as follows (PV (+)/ PV (-)); tsunami (35/ 33), evacuation area of the nuclear disaster (4/ 8), and other damages due to the earthquake (19/ 11).

**Table 1:** Parameters (glycemic control, lipid test) of PV (+), PV (-), and all the rest of diabetes patients other than PV (+) at SCH.

	Patients with disaster victim certificate		All the rest patients
	PV (+)	PV (-)	
Total n	58	52	575
Age	63.3 (9)	62.5 (11.7)	67.3 (14.1) *
M/F	38/ 20	28/ 24	335/ 240
E/T/N	19/35/4	11/33/8	NA
BG (mg/dl)	150 (37.7)	136 (57)	154.2 (50.7)
HbA1c (%)	7.3 (1)	7.4 (1.7)	7 (1.3) *
TC (mg/dl)	186.2 (37.9)	180.7 (33.3)	182.5 (33.4)
TG (mg/dl)	147 (85.4)	145.2 (85.2)	145.1 (88.3)
HDL-C (mg/dl)	53.1 (16.6)	53.4 (16.4)	51.8 (14.2)
cLDL-C (mg/dl)	103.6 (28.2)	98.3 (30)	101.9 (27.9)

Data are presented as the mean (SD).

*: p<0.05.

BG, blood glucose concentration; cLDL-C, calculated low-density lipoprotein cholesterol; F, female; HbA1c, glycosylated haemoglobin A1c; HDL-C, high-density lipoprotein cholesterol; M, male; NGSP, National Glycohemoglobin Standardization Program; TC, total cholesterol; TG, triglycerides

All participants had type 2 diabetes. Males were prevalent (65.5%) in the study sample. The median age of the subjects at the initial hospital visit after the January 2011 was 63.3 years. Table 2 presents other pre- and post-disaster characteristics of the subjects. Following the disasters, the mean values for BMI (p = 0.02), TC (p = 0.03), and cLDL-C (p<0.001) were significantly reduced and accompanied by a statistically significant increase in the mean HDL-C (p = 0.03). In contrast, the pre- and post-disaster mean values for BG (p = 0.9), triglycerides (p = 0.3), SBP (p = 0.9) and DPB (p = 0.8) did not significantly differ.

**Table 2:** Parameters (BMI, glycemic control, lipid tests and blood pressure) of all study subjects pre-disaster and post-disaster.

Total n	58		
Age	63.3 (9.0)		
M/F	38/20		
E/T/N	19/35/4		
	Pre-disaster	Post-disaster	*P*-value
BMI (kg/m^2^)	25.2 (4.1)	24.8 (4.2)	0.02*
BG (mg/dl)	149.1 (45.0)	148.1 (45.0)	0.9
HbA1c (%)	7.3 (1.0) (IFCC: 56)	7.1 (1.0) (IFCC: 54)	0.008*
TC (mg/dl)	183.3 (38.6)	175.7 (39.9)	0.03*
TG (mg/dl)	132.6 (85.6)	143.8 (86.4)	0.3
HDL-C (mg/dl)	51.5 (17.0)	54.2 (16.7)	0.03*
cLDL-C (mg/dl)	105.2 (30.9)	92.7 (30.8)	<0.001*
SBP (mmHg)	132.0 (14.5)	132.2 (17.0)	0.9
DBP (mmHg)	77.0 (8.7)	77.4 (10.6)	0.8

Data are presented as the mean (SD).

BG, blood glucose concentration; BMI, body mass index; cLDL-C, calculated low-density lipoprotein cholesterol; DBP, diastolic blood pressure; E, no. of patients whose house destroyed by the earthquake; F, female; HbA1c, glycosylated haemoglobin A1c; HDL-C, high-density lipoprotein cholesterol; M, male; N, evacuation area due to the Fukushima Nuclear Disaster; NGSP, National Glycohemoglobin Standardization Program; SBP, systolic blood pressure; T, the area hit by the Tsunami; TC, total cholesterol; TG, triglycerides

**Fig 2:**
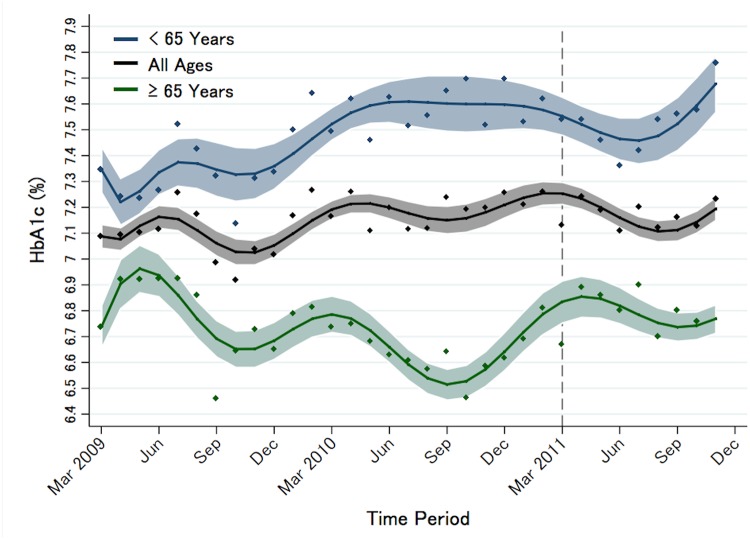
Changes in HbA1c levels from the pre- to post-disaster period. Patients were categorized by age: (A) all patients, (B) ≥65-years, and (C) <65 years.

Fig 2 shows the B-spline-smoothed trend for the HbA1c levels during the pre- and post-disaster periods with the 95% confidence bounds by age group: <65 years (upper), total population age (middle), and ≥65 years (lower).

The season-specific findings are described in Table 3. To investigate the seasonal variation in diabetes profiles, HbA1c levels in 2011 for each season were compared with the respective averages of the HbA1c levels in 2009 and 2010. Examining the data for the four groups stratified by age group and sex, no significant differences were observed in the mean HbA1c levels between the groups in all seasons of the year.

**Table 3:** Comparison of season-specific mean pre- and post-disaster HbA1c NGSP (%) followed by IFCC (International Federation of Clinical Chemistry) (mmol/mol) in parentheses.

		Pre-disaster	Post-disaster
Age group	Season*	Average of 2009 and 2010	2011
Males			
<65 years	Spring	7.6 (60)	7.4 (57)
	Summer	7.7 (61)	7.4 (57)
	Autumn	7.5 (58)	7.6 (60)
	Winter	7.8 (62)	7.8 (62)
≥65 years	Spring	6.9 (52)	6.8 (52)
	Summer	6.8 (51)	6.7 (50)
	Autumn	6.6 (49)	6.7 (50)
	Winter	6.8 (51)	6.7 (50)
Females			
<65 years	Spring	7.2 (55)	7.3 (56)
	Summer	7.2 (55)	7.1 (54)
	Autumn	7.1 (54)	7.4 (57)
	Winter	7.1 (54)	7.6 (60)
≥65 years	Spring	6.7 (50)	7.0 (53)
	Summer	6.6 (49)	6.8 (51)
	Autumn	6.5 (48)	6.7 (50)
	Winter	6.7 (50)	6.7 (51)

by age group and sex

*Spring: 11 March—10 June; Summer: 11 June—10 September; Autumn: 11 September—10 December; Winter: 11 December—10 March

Table 4 shows the changes in medication usage. DPP4-inhibitor was the only oral anti-diabetic agent exhibiting increased utilization, whereas utilization of pioglitazone decreased by 30.8%. Other drugs or therapies are too small in number to analyze statistically. The use of antihypertensive drugs increased after the disaster. However, the number of lipid-lowering agents administered did not change after the earthquake.

**Table 4:** Number of patient using diabetes treatment drugs, lipid-lowering drugs and antihypertensive drugs pre-disaster and post-disaster and percentage changes.

	Pre-disaster	Post-disaster	% Change
Hypoglycemic agents			
Sulfonylureas	24	23	-4.2
Glinides	2	1	-50
a-Glucosidase inhibitors	17	16	-5.9
Biguanides	24	24	0
Pioglitazone	13	9	-30.8
DPP-4 inhibitors	10	16	60
Insulin therapies	17	17	0
Dietary therapy only	3	2	-33.3
Lipid-lowering agents			
Statins	17	17	0
Fibrates	3	3	0
Antihypertensive agents			
ACEI	5	5	0
ARB	20	21	5
CCBs	22	24	9.1
Diuretics	11	11	0
Other	8	9	12.5

ACEI, angiotensin converting enzyme inhibitor; ARB, angiotensin II receptor blocker; CCB, calcium channel blocker; DPP-4, dipeptidyl peptidase-4

As shown in Table 2, Table 3, and Fig 2, HbA1c levels did not differ statistically despite the observed changes in medication usage. Conversely, with respect to lipid profiles, the mean LDL was reduced and the mean HDL was increased although no prescription changes were recorded. Anti-hypertensive drugs were utilized, but there was no significant difference in usage before and after the disaster.

## Discussion

In the era of the diabetes pandemic [18], adequate control of type 2 diabetes is essential for preventing complications. Previous studies have reported deterioration of glycemic control in patients with type 2 diabetes due to unhealthy lifestyles and mental distress [2]. In the aftermath of disasters, glycemic control in patients with type 2 diabetes is particularly difficult due to the presence of several factors potentiating deterioration. However, the optimal strategy for managing patients with type 2 diabetes during the period following a disaster has yet to be determined.

This study suggested that type 2 diabetes could be effectively managed through periodical visits to an outpatient clinic even in the aftermath of the disaster. Managing type 2 diabetes following a disaster was difficult for several reasons as noted in previous reports [6,19,20]. First, living in an evacuation shelter may result in low physical activity, as indicated in a previous report which suggested that mass urban disaster was associated with reduced levels of physical activity [21]. Second, the resulting changes in dietary patterns may lead to deterioration of glycemic control. In this situation, the consumption of meat might have been increased due to radio-contamination of seawater [22,23], whereas the former dietary pattern in this area was characterized by a high intake of fish and vegetable. Of note, previous studies suggest that a westernized dietary pattern is associated with a risk for type 2 diabetes [24,25]. Third, disasters place a great deal of stress on patients residing in the affected areas [26,27]. Despite the enormous impact of the disaster, this study suggested that the HbA1c did not increase significantly following the disaster in this disaster-affected cohort. The demographic characteristics of patients with periodical visits and patients without periodical visits shows no difference. Compared with all the rest diabetes patients who visit SCH, this cohort of 58 patients marks higher HbA1c. Regarding patients age, 58 patients are younger than the rest of all. Together with the previous report from Rikuzentakata city[12], this study cohort might be younger and HbA1c higher population. Periodical hospital visits may play a pivotal role in effectively managing diabetes during and after the disaster.

While medical institutions in the affected areas faced a critical shortage of healthcare providers, our hospital managed the outpatient clinic by allocating the staff adequately and through voluntary cooperation. SCH outpatient department maintained the same capacity of outpatient department as before, while inpatient capacity was temporally downsized. Although there was confusion promptly after the disaster, five full-time doctors and several part-time doctors, 50 nurses in total worked at SCH. Fifty to one hundred patients visited the outpatient department daily the same as before. While SCH were spared from tsunami, there ware only 200m from the inundated area. There were some affected staffs continued working from evacuation shelters. As SCH is 45km distant from the Fukushima Daiichi nuclear power plant and was not stratified into the mandatory evacuation area, some health care providers in the evacuation area due to the Fukushima Nuclear Disaster relocated to Soma city and supported our hospital.

Recently, empowerment by healthcare providers in addition to self-management by patients has been deemed to be essential to the treatment of diabetes [28]. In SCH, medical doctors performed personalized dietary instruction and voluntarily checked supplied lunch from evacuation shelters. Hospital staffs especially put emphasis on education about patients’ diet. Supplied bottled water was provided for diabetes patients at SCH to prevent dehydration. Monthly-lecture for diabetes was resumed in April 2011 and diabetes patients’ association committee was held in May. This report may provide useful information regarding the efficacy of empowerment by healthcare providers in controlling diabetes during the post-disaster period. Further investigation is necessary to clarify which factors contributed to this outcome as periodical hospital visits can offer multiple advantages, such as providing an adequate medication supply, adherence, mental support, lifestyle advice, as well as other benefits.

Regarding medical products, the required number of pharmaceuticals including insulin for patients was counted immediately after the disaster. Hospital staffs visited closed hospital in the mandatory evacuation area to be given pharmaceutical and medical products one week after the disaster. Fortunately, dispensing pharmacies and wholesalers of drug did not close and maintained a distribution of drugs. For patients who lost glucose test sensors, 22 sensors were lent from the hospital. SCH resumed complimentary shuttle service one month after the disaster. Telephone contacts to diabetes patients’ association members were performed two months after the disaster.

In the aftermath of the Fukushima Nuclear Disaster, Soma city local government continued to play a pivotal role in sustaining the healthcare system as well as healthcare providers. The local government initiated radiation exposure screening for the affected residents.[29–31] As far as the medical requisite materials were concerned, the local government officers visited Koriyama city, relatively close to Tokyo, to obtain relief supplies. Despite the immense damage, the local government rapidly arranged temporary housing, and all evacuation centers were closed on June 17, 2011. Adequate diets were also provided for the residents in the temporary housing. At least 9 of 58 patients were forced to live in temporary housings due to the total destruction of their house. Considering 7895 of total 79812 people lived in temporary housings in Soma district (Soma city and Minamisoma city in 2011), the population ratio of these 58 patients is assumed to be consistent with that of the stricken areas. Moreover, the municipal government constructed public housing so that the disaster victims could mutually assist one another and avoid social isolation. [16]

DPP4 inhibitors may be effectively administered to prevent deterioration in patients with type 2 diabetes in the aftermath of disasters. Diabetes doctors at our hospital willingly prescribed DPP4 inhibitors to avoid hypoglycemia. Our findings showed that the number of DPP4 inhibitors used had markedly increased. Previous reports have suggested that patients treated with DPP4 inhibitors have a low risk for hypoglycemia even under irregular eating patterns. This is due to the fact that DPP4 inhibitors stimulate insulin release and inhibit glucagon secretion in a glucose-dependent manner [32,33]. Thus, DPP4 inhibitors may be strong candidates for medications used to manage diabetes in the immediate aftermath of a disaster as well as in the more remote period following the disaster.

This study revealed that HbA1c levels did not significantly differ between the periods before and after the disaster in either the elderly or the younger population. While previous reports suggested that HbA1c levels fluctuate throughout the year [34], our findings imply that periodical hospital visits may adequately control type 2 diabetes even in the aftermath of the disaster as well as in daily clinical practice. For more precise management of type 2 diabetes during and after the disaster, personalized intervention is needed to improve disease control in the elderly population.

Several factors may limit the ability to generalize the results of this study. First, the small cohort, short follow-up period, and retrospective analysis might have limited statistical power. Second, the lack of detailed data made it difficult to accurately estimate the impact of the disaster. While we performed season-specific analyses of HbA1c changes, adjustments were not performed because it was difficult to obtain data on other parameters, such as body weight before the disaster. Third, it is not clear whether this cohort truly represents the average pattern of affected residents. However, we could not obtain the rest of individual data due to personal information protection law.

## Conclusion

Patients with type 2 diabetes who participated in periodical hospital visits did not show significant deterioration in HbA1c levels, indicating that periodical hospital visits may provide adequate disease control.

## Supporting Information

S1 Permission Esri permission. (PNG)
