# Supplementary figures and images for: Managing Type 2 Diabetes Mellitus through Periodical Hospital Visits in the Aftermath of the Great East Japan Earthquake Disaster: A Retrospective Case Series
Source: PLoS One. 2015 May 6;10(5):e0125632. doi: 10.1371/journal.pone.0125632 (PMC4422625; doi:10.1371/journal.pone.0125632)

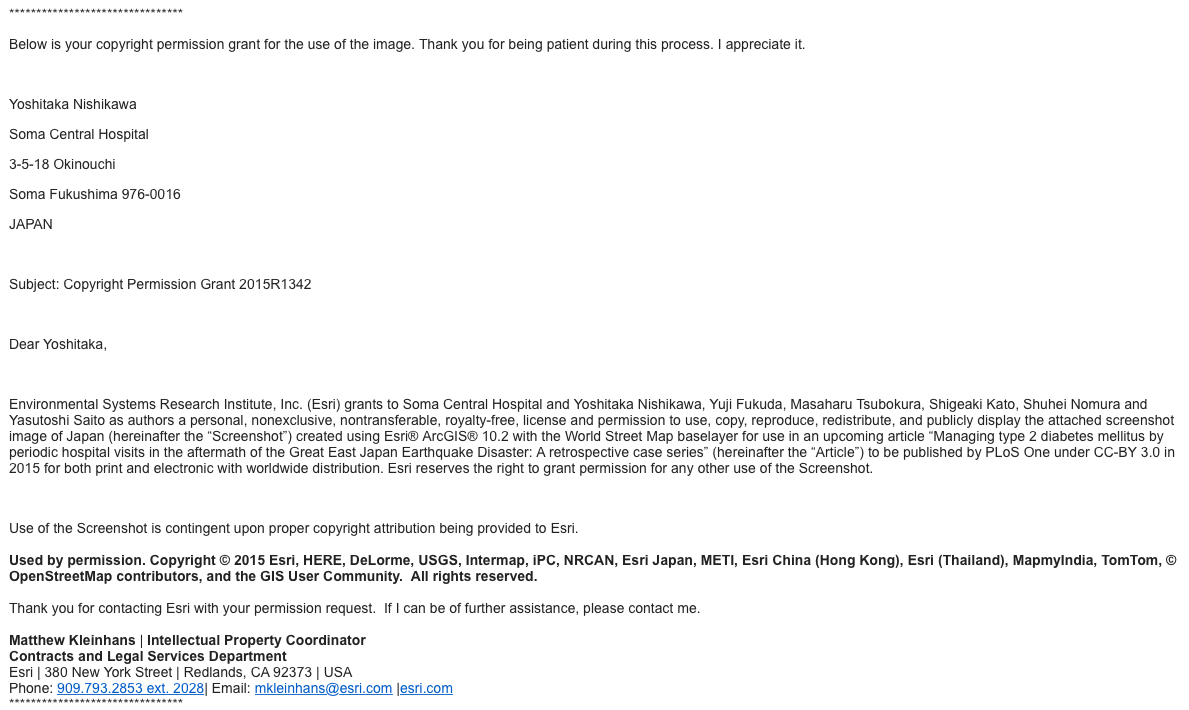

Supplement: S1 Permission — (PNG) [file pone.0125632.s001.png]
